# Probing the Role of Cysteine Thiyl Radicals in Biology: Eminently Dangerous, Difficult to Scavenge

**DOI:** 10.3390/antiox11050885

**Published:** 2022-04-29

**Authors:** Bernd Moosmann, Parvana Hajieva

**Affiliations:** 1 Evolutionary Biochemistry and Redox Medicine, Institute for Pathobiochemistry, University Medical Center of the Johannes Gutenberg University, 55128 Mainz, Germany; 2 Institute for Translational Medicine, MSH Medical School Hamburg, 20457 Hamburg, Germany

**Keywords:** aging, chain-transfer agent, cysteine persulfide, glutathione, lipid peroxidation, longevity, peroxyl radical, selenocysteine

## Abstract

Thiyl radicals are exceptionally interesting reactive sulfur species (RSS), but rather rarely considered in a biological or medical context. We here review the reactivity of protein thiyl radicals in aqueous and lipid phases and provide an overview of their most relevant reaction partners in biological systems. We deduce that polyunsaturated fatty acids (PUFAs) are their preferred reaction substrates in lipid phases, whereas protein side chains arguably prevail in aqueous phases. In both cellular compartments, a single, dominating thiyl radical-specific antioxidant does not seem to exist. This conclusion is rationalized by the high reaction rate constants of thiyl radicals with several highly concentrated substrates in the cell, precluding effective interception by antioxidants, especially in lipid bilayers. The intractable reactivity of thiyl radicals may account for a series of long-standing, but still startling biochemical observations surrounding the amino acid cysteine: (i) its global underrepresentation on protein surfaces, (ii) its selective avoidance in aerobic lipid bilayers, especially the inner mitochondrial membrane, (iii) the inverse correlation between cysteine usage and longevity in animals, (iv) the mitochondrial synthesis and translational incorporation of cysteine persulfide, and potentially (v) the ex post introduction of selenocysteine into the genetic code.

## 1. Introduction: Amino Acid Redox Activity

Redox reactivity has played a major role during the stepwise evolutionary fixation of the 20 canonic, proteinogenic amino acids [1]. Quantum chemical analysis of the standard amino acids against the background of diverse alternative, abiotic amino acids has indicated that the last six amino acids were in fact introduced into the genetic code because of their innovative redox properties [2,3]. This is in clear contrast to the first 14 amino acids, which were evidently selected for their protein structure-building properties [1,3,4,5,6,7,8], comprising a set of compounds with carefully selected sizes, shapes, and hydrophobicities [9,10,11].

Redox reactivity is obviously a useful chemical property for metabolic and catalytic purposes. Moreover, it is hard to refute that redox reactions were among life’s very earliest biochemical transformations [12,13,14]. Still, targeted enzymatic reactions in modern cells are generally catalyzed by locally bound, redox-active cofactors [15,16]. These are usually derived from nucleotides (e.g., NADH) and may have been taken over from an earlier RNA world [17,18], or they reflect the primordial geochemistry of the site where life may have originated (e.g., FeS clusters) [3,13]. Active-site redox catalysis by genetically encoded amino acids does also exist, but it is less widespread and often depends on the concomitant presence of the ancient nucleotide or metal cofactors [19,20]. Cysteine-dependent oxidoreductases may be viewed as exceptions, but even here, one or two redox-active residues in a specific site of the protein (the “catalytic center”) are sufficient for catalysis [21]. Hence, the question arises why genetic code alterations should have occurred that resulted in a quantitative introduction of high numbers of redox-active components (i.e., the new amino acids) into proteins far away from the catalytic centers.

The very likely answer to this conundrum is molecular oxygen [1,2,3]. The rise of this molecule visibly triggered a whole plethora of compensatory responses to offend all types of harmful oxidative side reactions within the catalytic centers and on protein surfaces. The aim was to sustain the functionality of the inherited enzyme machinery, which had initially been developed under anoxic conditions. Two prominent examples for the usefulness of protein redox activity even far away from catalytic centers are (i) the interference with protein surface oxidation by reactive oxygen species (ROS), especially in membrane proteins, and (ii) the prevention of catalytic center self-destruction by highly oxidizing electron holes, as sketched in the following.

The late amino acid methionine, when surface-exposed, is involved in an antioxidant catalytic cycle of oxidation (to methionine sulfoxide) and reduction (back to methionine) [22,23]. For example, respiratory chain complexes in animals feature a massive enrichment of methionine [24], which strongly correlates with aerobic metabolic rate [25]. This enrichment is effectuated by alterations in the mitochondrial genetic code that have occurred several times independently during the Cambrian radiation [26,27]. Notably, the latter has widely been linked to rising biospheric oxygen levels [28,29]. Even without genetic code alterations, methionine in animals tends to be accumulated in the vicinity of sites of high ROS production, or in proximity to particularly susceptible targets, such as single-stranded nucleic acids [25].

Tyrosine and tryptophan are also amply involved in protective oxidation–reduction cycles such as that of methionine, but with two major differences: they are usually oxidized by a single electron and without the addition of an oxygen atom. Moreover, their reactions often occur in the interior of a protein. Perhaps the most striking example is the transfer of single electrons (or electron holes in the opposite direction) along appropriately positioned chains of tyrosine and tryptophan residues [30]. Long-range electron transfer through such chains can be part of primary catalysis, but may also prevent the fatal auto-oxidation of an enzyme in the case of substrate deficiency [30,31]. In addition, it may suppress ROS production as a side reaction [32], and it may repair cofactor oxidation by ambient oxygen [30,33]. Suitable chains have been identified in numerous classes of enzymes [34], indicating that the oxidizing power of oxygen could not have been safely exploited without genetically encoded tyrosine and tryptophan [32].

On the surfaces of membrane proteins, tyrosine and tryptophan are involved in a protective cycle of oxidation by lipid peroxyl radicals, following reduction by intramembrane (tocopherol) or membrane surface (ascorbate) reductants [3,35,36]. In small, unfolded peptides, both amino acids may exhibit similar activities in the aqueous space [37]. As with methionine, but somewhat less pronounced, a general accumulation of these residues in ROS-susceptible sites has been noted [1,35,36].

The idea that the last amino acids’ addition to the genetic code was triggered by biospheric oxygenation [1,2,3] has recently found support by a series of unexpected findings: first, the evolution of the oxygen-producing photosystem II in cyanobacteria had to be significantly predated [38,39,40,41], virtually closing the temporal gap between both processes. Second, antioxidant enzyme evolution in cyanobacteria (i.e., superoxide dismutases, SODs) [42,43] as well as the evolution of oxygen-utilizing enzymes [38,43] have arguably taken place much earlier than traditionally assumed. Thereby, genetic code finalization and oxygen-dependent ROS production have come closely together on the evolutionary time scale. These findings have been backed by revised geochemical oxygen records that have made early biospheric oxygen production possible, if not likely [38,44,45,46,47]. In fact, environmental peroxide (i.e., ROS) production may have even been effectuated by geochemical processes [47,48]. After all, compelling modern precedents are known regarding genetic code alterations aimed at introducing or increasing redox-active amino acids in preexisting proteomes, namely methionine [3,24] and selenocysteine [49,50].

A key criterion for the adaptive, potentially antioxidant introduction of the recent amino acids methionine, tyrosine, and tryptophan has been their local accumulation at critical sites of oxidative damage (as in mitochondria), or their global accumulation in aerobic versus anaerobic life forms [1,3,24,25,34,36,51]. What about cysteine in this respect?

## 2. Cysteine Redox Activity: Utterly Convenient, but with a Dark Side: Thiyl Radicals

The conventional view of cysteine, the arguably most redox-reactive amino acid [52,53], would be that it has provided a plethora of useful, mostly redox-dependent properties after its surprisingly late introduction into the genetic code [3,54,55]. Prominent examples for such useful redox properties would be the establishment of disulfide bridges [56], the anchorage of metal cofactors [3,57], or the formation of catalytic redox relay systems such as the thiol-dependent peroxidases [21,58]. Nevertheless, it has come with true surprise that in essentially every biological system that has been investigated accordingly, cysteine seems to be avoided when a heightened danger of oxidation exists. For instance, cysteine is underrepresented in aerobic versus anaerobic bacteria [3,51,59,60], archaea [3,59], and unicellular eukaryotes [3,59]. It is avoided in mitochondria [59], and within the mitochondria, it is particularly avoided in the respiratory chain [61], but only in aerobic species that permanently use their respiratory chain. Anaerobic–parasitic helminths do not deplete cysteine in their respiratory chain, which they utilize only during their short free-living stage [59,61]. Within the respiratory chain, highly expressed proteins avoid cysteine more stringently than average proteins [61]. In aerobic animals, the degree of cysteine avoidance is arguably the best available molecular predictor of longevity [59,62]. What to do with all this biological information in view of the numerous cysteine oxidative modifications and reactive sulfur species [52,53,63] that might underlie it?

A few specific observations have given relevant hints at which cysteine reactivities might be the drivers of evolutionary cysteine depletion. Frist, solitary surface cysteines are generally avoided in all branches of life, which is not the case for paired cysteine residues [64]. Thus, cysteine has been classified as an either functional or detrimental, but rarely neutral building block in proteins [64,65]. Second, evolutionary cysteine depletion is most pronounced in transmembrane domains [3,61], whereas the pattern of cysteine avoidance in aqueous proteins (i.e., on the surface) indicates that cysteine is not per se disfavored in hydrophobic phases such as protein interiors [64]. Hence, the chemical interaction of transmembrane cysteine residues with surrounding lipids emerges as a prime suspect. Third, methionine’s redox chemistry clearly must not share the sought-after, adverse redox property of cysteine because it is highly enriched under many conditions where cysteine is depleted [24]. This effectively excludes the involvement of reactions adding an oxygen atom to cysteine’s sulfur atom [62]. In summary, the conclusion was reached [62] that the dark side of protein cysteine might be hidden in a single thiol redox reaction that would be especially prominent in hydrophobic phases (thus not involving highly polar intermediates), that would not be alike to any known reaction of methionine, and that would arguably not add oxygen in order to be reversible without enzymatic catalysis. Thus, the reversible formation of thiyl radicals was postulated to chiefly account for the evolutionary patterns of cysteine depletion [62].

Thiyl radicals have been demonstrated to elicit a wide spectrum of potentially adverse biochemical reactivities, ranging from the induction of lipid peroxidation [66] and fatty acid cis-/trans-isomerization [67] to L-/D-amino acid isomerization [68] and peroxidative protein backbone cleavage [69]. Very recent work indicates that beyond these reactivities, intramembrane thiyl radicals also act as catalytic accelerators of already initiated, ongoing lipid peroxidation chain reactions in vivo [70,71]. According to this mechanism, thiyl radicals in living systems would act as chain-transfer agents, which is the very same function for which they have been employed in technical chemistry for more than 80 years [72,73]. In the following, we summarize the essential biochemistry of thiyl radicals in more detail, to probe whether their unique reactivity might indeed be dangerous enough to enforce evolutionary cysteine depletion.

## 3. Thiyl Radicals: Formation and Reactivity

The reactivity of thiyl radicals has foremost been studied using in vitro systems amenable to pulse radiolysis experiments [66,67,68,74,75,76]. More direct and specific measurement of transiently formed thiyl radicals in intact biological systems has remained an intricate challenge [77]. Still, a widely accepted signature chemistry has been elaborated, which is the formation of (mono)unsaturated trans-fatty acids from native cis-fatty acids [67,78,79,80,81]. 

**Figure 1:**
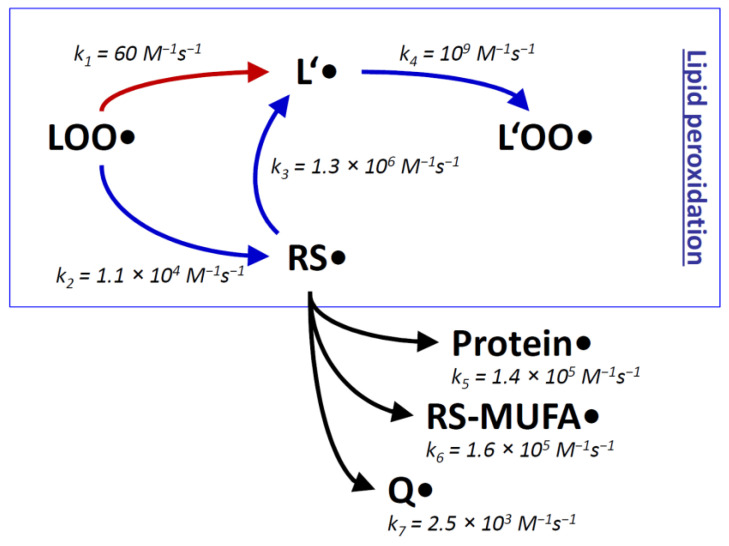
Rate constants k of lipid peroxidation with and without chain-transfer catalysis by thiyl radicals. During the lipid peroxidation chain reaction (boxed), lipid peroxyl radicals LOO^●^ induce the continuing formation of other lipid peroxyl radicals L’OO^●^ via slow radicalization of native lipids L’ (k_1_), followed by fast addition of ambient oxygen (k_4_). The slow radicalization process k_1_ can be detoured by chain-transfer catalysis, which involves two steps: moderately fast radicalization of thiol groups towards thiyl radicals RS^●^ (k_2_), followed by rapid thiyl radical attack on native lipids L’ (k_3_). Since k_2_ is substantially higher than k_1_, the overall chain reaction is accelerated by approximately the ratio of k_2_/k_1_. Some alternative reactions of thiyl radicals with proteins, monounsaturated fatty acids (MUFAs), and ubiquinone (Q) are indicated (k_5_-k_7_). All of these are slower than the propagation of the radical towards native lipids L’. The provided rate constants represent: k_1_, reaction of peroxyl radicals with linoleic acid [83]; k_2_, reaction of peroxyl radicals with cysteine in nonpolar solution [82]; k_3_, reaction of cysteine radicals in nonpolar solution with linoleic acid [66]; k_4_, reaction of lipid radicals with oxygen [83]; k_5_, reaction of cysteamine radicals with serine (the fastest-reacting amino acid in proteins) [75]; k_6_, reaction of mercaptoethanol radicals with methyl oleate [79]; k_7_, reaction of cysteine radicals with ubiquinol-0 [84]. Modified from [70].

The inevitable formation of thiyl radicals in vivo can be rationalized in terms of the very rapid reaction of certain trigger radicals with thiol groups, for example in the reactions initiated by HO^•^ (k = 6.8 × 10^9^ M^−1^s^−1^ [76]) and H^•^ (k = 1.7 × 10^9^ M^−1^s^−1^ [76]). Also rather fast are the corresponding reactions of alkoxyl radicals (for Me_3_CO^•^: k = 6.6 × 10^7^ M^−1^s^−1^ [82]) and carbon-centered radicals (for Me_2_C^•^H: k = 6.7 × 10^6^ M^−1^s^−1^ [82]). Peroxyl radicals, however, react relatively slowly with alkyl thiols (for R_2_CHOO^•^: k = 4.2 × 10^3^ M^−1^s^−1^ [82]). Still, during lipid peroxidation, the attack of an intramembrane peroxyl radical ROO^•^ on a thiol RSH is at least one order of magnitude faster than the canonic reaction, which would be the attack of the peroxyl radical ROO^•^ on a native bisallylic position of a polyunsaturated lipid L’ (Figure 1) [70]. The latter reaction is rate-limiting for the propagation step of lipid peroxidation, and it may even be rate-limiting for biological lipid peroxidation as a whole under certain conditions [70,83]. The transiently formed thiyl radicals RS^•^ then attack polyunsaturated lipids L’ very efficiently. Hence, thiol groups in molecular contact with polyunsaturated lipids, specifically intramembrane thiols groups of membrane proteins, may possess the detrimental capacity to accelerate lipid peroxidation by acting as chain-transfer catalysts. Chain-transfer catalysis shunts the rate-limiting step of an ongoing lipid peroxidation chain reaction (Figure 1) [70,71,72,73].

In recent experimental tests of this hypothesis, living cells and *Caenorhabditis elegans* nematodes were administered with lipophilic thiol compounds to mimic the effects of intramembrane protein thiol groups. In fact, a so far novel type of prooxidative biochemistry was observed [70,71], which was characterized by accelerated lipid peroxidation at unchanged rates of initiation, extensive damage to membrane proteins, loss of PUFAs, trans-fatty acid formation and a reduction of organismal lifespan [70]. Tumor cells, which are widely assumed to have higher initiation rates than most other cells [85,86], were particularly susceptible to the cytotoxic effects of lipophilic thiols [71].

**Figure 2:**
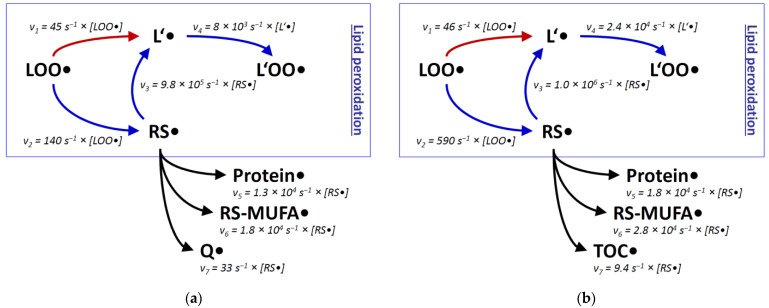
Reaction rates v of lipid peroxidation estimated for two cellular compartments with and without involvement of thiyl radicals. Relative rates v were determined with the second-order rate law v = k × [S_1_] × [S_2_], where k is the reaction rate constant (taken from Figure 1), [S_1_] the concentration of the native substrate, and [S_2_] the concentration of the attacking radical. Specific values for [S_1_] were adopted from the literature as detailed in Table 1, with (**a**) reflecting the concentrations in the inner mitochondrial membrane of rat liver hepatocytes, and (**b**) representing generalized rat liver membranes. Specific values for [S_2_] are unknown and may be rather variable, depending on the physiological state of the cell. Still, within each panel, velocities involving the same radical can be directly compared, i.e., v_1_ with v_2_ and v_3_ with v_5_-v_7_. Abbreviations are used as in Figure 1; TOC is α-tocopherol. (**a**), modified and expanded from [70].

Rate constants alone can be misleading in the discussion of molecular fates, especially when reaction partners with large concentration differences are involved. To better approximate the reactivity of a once formed thiyl radical in biological systems, we have also assessed the molar concentrations of potential reactants. Via kinetic rate laws, these concentrations can provide estimates of the true reaction rates v to be expected in vivo [70]. For the present discussion, we have recapitulated and expanded the published calculations [70], which now encompass two well-established biochemical model systems: rat liver mitochondria, and generalized rat liver tissue. Rat liver mitochondria were analyzed separately for the inner membrane compartment and the total aqueous compartment; rat liver tissue was surveyed separately for the total membrane compartment and the total aqueous compartment (Figure 2, Table 1). For several compounds, plausible approximations had to be adopted, which are justified in the legend of Table 1.

When comparing the predictions about lipid peroxidation made from plain rate constants k (Figure 1) with approximated reaction rates v (Figure 2), the initial preponderance of the thiyl radical shunt (by a factor of ~20) becomes smaller, because intramembrane thiol concentrations are generally lower than the concentrations of polyunsaturated lipids [70]. Nevertheless, an advantage remains for the thiyl radical-catalyzed reaction sequence in both membranes, namely in the inner mitochondrial membrane, and in pooled non-mitochondrial liver membranes (by a factor of ~2 and ~10, respectively). Still, ambiguity comes from the unknown degree of surface exposure of intramembrane cysteine residues (estimated to be ~20% [70]), which was not considered here. Similarly disregarded were all other factors that might impair the kinetic activities of the compounds under consideration, such as protein binding. Finally, thiol group covalent modifications such as sulfuration [106] could be relevant and prevent thiyl radical formation and thus chain-transfer catalysis [107], but had to be neglected here nevertheless.

Regarding alternative reaction pathways of intramembrane thiyl radicals, the consideration of reactant concentrations (Figure 2) did not substantially alter the conclusions from plain rate constants (Figure 1). Protein side chains as well as MUFAs react less efficiently with thiyl radicals than bisallylic positions of polyunsaturated lipids (by a factor of 10-50). Concerning the reaction with protein side chains, however, substantial uncertainty comes from the fact that protein surface thiyl radicals would be ideally located to react with neighboring amino acid side chains [75,88]. This effect could increase the relative importance of the protein damage pathway versus the lipid peroxidation pathway. Comparable trade-off effects between lipid and membrane protein damage have been noted before [36]. The experimental observation that chain-transfer catalysis by lipophilic thiols evokes membrane protein damage [70] cannot be readily assigned to a specific mechanism because lipid peroxidation in itself also generates protein-damaging species, such as reactive aldehydes [83].

## 4. Strategies of Anti-Thiyl Radical Defense: Scavenging

Inspecting the chemical options of intramembrane thiyl radicals more comprehensively (Table 1), it becomes evident that the attack on polyunsaturated lipids and thus the catalysis of lipid peroxidation is the fastest reaction to occur, with a relative reaction rate of ~10^6^ s^−1^. This high velocity is the result of a high rate constant (1.3 × 10^6^ M^−1^s^−1^) combined with a very high substrate concentration (750 mM in the inner mitochondrial membrane), which none of the other investigated competitors can outcompete. The four (relatively reactive [75]) amino acids evaluated here exhibited lower relative reaction rates and concentrations irrespectively of the membrane type studied. Oxygen, in turn, reacts very quickly, but is only present at low micromolar concentrations. 

Notably, the major phenolic antioxidants in both investigated membrane compartments, ubiquinone (Q) and α-tocopherol (TOC), also cannot play a role as scavengers of thiyl radicals (Table 1). Both Q and TOC are insufficiently reactive [76,78,84], and TOC is also too diluted under physiological conditions to interfere with the attack of thiyl radicals on PUFAs, a reaction that ends up to be at least 10^4^-fold faster. Still, as both compounds exhibit rate constants in the range of 10^6^ M^−1^s^−1^ [84] for their reaction with peroxyl radicals, which is their evolutionarily selected function [2,108,109], they are arguably involved in the restriction of thiyl radical formation in the first place.

The irrelevance of phenolic antioxidants as thiyl radical scavengers appears to be a general phenomenon that also applies to aqueous polyphenols such as flavonoids. Chemical calculations have shown that flavonoids rarely exhibit corresponding rate constants higher than 10^4^ M^−1^s^−1^ [84,110]. Together with the limited concentrations that are attainable by flavonoids in vivo (e.g., peak concentrations of less than 1 µM quercetin were observed even after supplementation [94]), extremely low reaction rates result (Table 1). Experimental studies in biomimetic micelle models and in solution have confirmed that even the best peroxyl radical-scavenging phenols such as TOC are indeed poor antioxidants for thiyl radicals [76,78,111].

In contrast to the largely unreactive phenolic antioxidants, isoprenoid polyenes such as carotene and lycopene are known to exhibit very high rate constants (~10^9^ M^−1^s^−1^) regarding their reaction with thiyl radicals [74]. Still, even under conditions of excessive supplementation (yielding 10^4^-fold higher concentrations than without supplementation) [96] and when examining the very tissue in which dietary lycopene accumulates (the liver) [102], the calculated relative reaction rate just leveled out with the relative reaction rate of lipid peroxidation (Table 1). Hence, serious doubts remain as to whether isoprenoid polyenes act as thiyl radical scavengers under real-world conditions.

To close the thiyl radical scavenger discussion more positively, the situation is clearly less bleak in the aqueous space. Embodied by ascorbate and glutathione, there are two widely distributed, millimolar concentrated low-molecular weight antioxidants that feature high rate constants (>10^8^ M^−1^s^−1^), resulting in reasonably high relative reaction rates (Table 1). In fact, both predicted rates closely resemble each other in the two modeled systems, and they are at least somewhat higher than the calculated reaction rates with proteinogenic amino acids. It arguably depends on the precise circumstances which of the two antioxidants performs more efficiently as a thiyl radical scavenger [100].

In summary, we propose that thiyl radical-specific antioxidants cannot exist in highly unsaturated lipid bilayers for simple kinetic reasons, and there is arguably only incomplete suppression of thiyl radical activity by ascorbate and glutathione in aqueous systems. In consequence, the suspicion arises that this kinetic impossibility has led to the widely observed phenomenon of protein thiol avoidance, especially in membranes. In this context, protein thiol avoidance encompasses more than just plain cysteine avoidance, as it may also come as cysteine modification (by sulfuration and other types of chemical derivatization), cysteine replacement (by selenocysteine), or cysteine reactivity diversion (towards glutathione, which exhibits similar reactivity, but less detrimental consequences of oxidation). These potential manifestations of protein thiol avoidance are briefly explored in the following.

## 5. Strategies of Anti-Thiyl Radical Defense: Cysteine Avoidance and More

Thiol-modifying persulfides are formed in ample amounts in mitochondria by cysteinyl-tRNA synthetase (CARS2) [112,113]. Persulfides can be readily transformed into perthiyl radicals (RSS^•^), which are yet much more stable than thiyl radicals and very likely unable to sustain chain-transfer catalysis [107,113]. Moreover, cysteine persulfide has been shown to be translationally incorporated into newly synthesized proteins [112,114]. Could these observations be interpreted as to represent a specific type of mitochondrial cysteine avoidance?

The mitochondrially encoded proteome of long-lived animals is exceptionally depleted of cysteine, with 22 remaining residues in 3786 amino acids (~0.58%) in humans, where approximately 100 residues would have been expected from nuclear coding patterns (~2.65%) [59]. Only four of these cysteine residues are conserved in animals, of which three are already known to have an essential function [59,115], whereas all other amino acids count between 18 and 127 conserved positions (mean: 47). In other words, there appears to be extreme pressure to avoid cysteine residues in the mitochondrially encoded proteome of aerobic, long-lived animals [59,61]. Thus, the selective synthesis of most cellular cysteine persulfide by mitochondrial cysteinyl-tRNA synthetase [112,114] may plausibly constitute an effort to cotranslationally insert benign cysteine persulfide instead of malign cysteine into the inner mitochondrial membrane. Notably, cells with inhibited mitochondrial cysteine persulfide production demonstrated significant defects in mitochondrial structure and respiratory chain function [112,113,114]. Admittedly, other interpretations may also account for the latter observations [114].

Nevertheless, substantial support for the thiol avoidance hypothesis comes from the consideration of the longevity effects of mitochondrial cysteine depletion: *C. elegans* treated with hydrogen sulfide (H_2_S) donors that increase persulfide formation [106,116] have been shown to exhibit an antioxidant longevity phenotype [117,118]. Conversely, micromolar concentrations of lipophilic thiols in the food of *C. elegans* have been found to entail a prooxidative, severely life-shortening effect [70]. After all, the specific acceleration of radical propagation (rather than radical initiation or termination) by thiyl radicals may explain why conventional attempts of life extension with antioxidants have failed consistently: they have only targeted radical initiation or termination, which are arguably not rate-limiting for aging [119,120].

Selenocysteine is a non-canonic proteinogenic amino acid that is incorporated into a small number of selenoproteins in specific, functionally relevant sites by alternative interpretation of certain UGA codons during translation [49]. In most cases, only a single selenocysteine residue is encountered, which is located in the catalytic center of an enzyme with oxidoreductase activity [49]. The use of selenocysteine instead of cysteine in the affected enzymes has not yet found an entirely convincing explanation, as the initially suspected and occasionally demonstrated catalytic advantage of selenocysteine is not universal [121,122]. For instance, it does not apply to thioredoxin reductase [121], the single selenoenzyme for which *C. elegans* maintains a complete selenocysteine insertion machinery [123]. An intriguing alternative hypothesis has argued that the deployment of catalytic selenocysteine might primarily serve the function to avoid circumstantial thiyl radical formation on the otherwise inevitable catalytic cysteine [122,124]. Thereby, the rapid self-destruction of the enzyme would be prevented [124,125], which is an argument that surprisingly resembles the self-protection of high-potential metalloenzymes by chains of tyrosine and tryptophan [34].

Glutathione is an ancient and widely distributed thiol compound [126] that shares with ascorbate, a compound biosynthetically restricted to photosynthetic organisms [127], a high reactivity towards thiyl radicals [75,100] (Table 1). The first intermediate in the reaction of glutathione with thiyl radicals is a relatively stable disulfide radical that may follow different downstream pathways, some of which involve the production of other toxic radicals such as superoxide [100]. Hence, ascorbate may often be more expedient in the safe disposal of thiyl radicals than glutathione [100]. Nevertheless, as a highly concentrated, low-molecular weight thiol in the aqueous space [87,93], glutathione may still be critically involved in the prevention and overall reduction of thiyl radical-mediated damage even when transiently transformed into a thiyl radical itself. First, in reacting with radicals formed in the aqueous space, glutathione may preclude their diffusion into the membrane compartment, where thiyl radical formation would be much more detrimental as detailed before. Second, considering just the aqueous space, the formation of a glutathione thiyl radical may be clearly preferred over the formation of a protein thiyl radical, because the maximum adverse outcome, i.e., the complete functional loss of the molecule, is much smaller in the case of glutathione: the tripeptide glutathione can be replaced at a substantially lower biosynthetic cost than an average 500-amino acid protein. Moreover, proteins may gain toxic functions such as hydrophobic aggregation properties during thiyl radical-mediated oxidation, which is arguably not the case with glutathione. Thus, the oxidation of glutathione instead of protein cysteine certainly represents a favorable outcome when a cell is confronted with thiyl radical-inducing oxidants.

Glutathione may have actually been shaped by evolution for the detoxification of thiyl radicals [3]. Intramolecular thiyl radical transfer enabled by the unusual γ-carboxyl linkage provides a tentative explanation [128,129] as to why the tripeptide γE-C-G is the major soluble thiol in most forms of life [126], instead of cysteine or another simple thiol. A specific role of glutathione in protein thiyl radical scavenging is furthermore suggested by the observation that prokaryotic clades possessing glutathione biosynthetic capacity generally do not exhibit lower proteomic cysteine contents under aerobic (versus anaerobic) conditions, which is in contrast to bacteria lacking glutathione [3].

## 6. Conclusions

The study of molecular evolution has provided ample evidence that surface-exposed, solitary thiol groups in proteins are highly problematic, epitomized by the universal avoidance of cysteine in aerobic membrane proteins. Closer inspection indicates that in many, and perhaps most cases, detrimental thiyl radical formation may underlie the avoidance of cysteine. The use of selenocysteine in critical sites, the replacement of cysteine by cysteine persulfide, and the maintenance of high and competing levels of less dangerous non-protein thiols such as aqueous glutathione may all be interpreted as to prevent protein thiyl radical formation. The adverse effects of intramembrane thiol groups on longevity in animals suggest that thiyl radicals are the prime radical species to accelerate organismal aging. For a complete overview of the multifaceted chemistry of thiyl radicals, the reader is referred to an authoritative review article [130].

## Figures and Tables

**Table 1:** Rate constants and relative reaction rates of thiyl radicals RS^•^ with cellular substrates in different compartments.

	Rate Constant k	Concentration c ^1^	Reaction Rate v_rel_ (= v/[RS^●^])
**Mitochondria**, **Aqueous Compartment**			
Ascorbate	6 × 10^8^ M^−1^s^−1^ [75]	5.6 × 10^−4^ M [87]	3.4 × 10^5^ s^−1^
Glutathione ^2^	1 × 10^8^ M^−1^s^−1^ [75]	3.9 × 10^−3^ M [87]	3.9 × 10^5^ s^−1^
Methionine	8 × 10^3^ M^−1^s^−1^ [75]	9 × 10^−2^ M [70]	7.2 × 10^2^ s^−1^
Phenylalanine	1.2 × 10^4^ M^−1^s^−1^ [75]	1.3 × 10^−1^ M [70]	1.6 × 10^3^ s^−1^
Serine	1.4 × 10^5^ M^−1^s^−1^ [75]	2.5 × 10^−1^ M [70]	3.5 × 10^4^ s^−1^
Threonine	4.6 × 10^4^ M^−1^s^−1^ [75]	1.9 × 10^−1^ M [70]	8.7 × 10^3^ s^−1^
Oxygen	2.2 × 10^9^ M^−1^s^−1^ [88]	2 × 10^−6^ M [89]	4.4 × 10^3^ s^−1^
**Mitochondria**, **Inner Membrane**			
MUFA ^3^	1.6 × 10^5^ M^−1^s^−1^ [79]	1.1 × 10^−1^ M [90]	1.8 × 10^4^ s^−1^
PUFA ^3,4^	1.3 × 10^6^ M^−1^s^−1^ [66]	7.5 × 10^−1^ M [90]	9.8 × 10^5^ s^−1^
Ubiquinol ^5^	2.5 × 10^3^ M^−1^s^−1^ [84]	1.3 × 10^−2^ M [91]	3.3 × 10^1^ s^−1^
α-Tocopherol ^6^	6.2 × 10^4^ M^−1^s^−1^ [84]	6.4 × 10^−5^ M [92]	4.0 × 10^0^ s^−1^
Methionine	8 × 10^3^ M^−1^s^−1^ [75]	7.9 × 10^−2^ M [70]	6.3 × 10^2^ s^−1^
Phenylalanine	1.2 × 10^4^ M^−1^s^−1^ [75]	1.2 × 10^−1^ M [70]	1.4 × 10^3^ s^−1^
Serine	1.4 × 10^5^ M^−1^s^−1^ [75]	9.6 × 10^−2^ M [70]	1.3 × 10^4^ s^−1^
Threonine	4.6 × 10^4^ M^−1^s^−1^ [75]	1.0 × 10^−1^ M [70]	4.6 × 10^3^ s^−1^
Oxygen	2.2 × 10^9^ M^−1^s^−1^ [88]	8 × 10^−6^ M [89]	1.8 × 10^4^ s^−1^
**Liver tissue**, **Aqueous Compartment**			
Ascorbate	6 × 10^8^ M^−1^s^−1^ [75]	1.3 × 10^−3^ M [87]	7.8 × 10^5^ s^−1^
Glutathione ^2^	1 × 10^8^ M^−1^s^−1^ [75]	7.2 × 10^−3^ M [93]	7.2 × 10^5^ s^−1^
Quercetin ^7,8^	4.0 × 10^3^ M^−1^s^−1^ [84]	<1.0 × 10^−6^ M [94]	< 1.0 × 10^−3^ s^−1^
Methionine	8 × 10^3^ M^−1^s^−1^ [75]	3.9 × 10^−2^ M [70]	3.1 × 10^2^ s^−1^
Phenylalanine	1.2 × 10^4^ M^−1^s^−1^ [75]	6.2 × 10^−2^ M [70]	7.4 × 10^2^ s^−1^
Serine	1.4 × 10^5^ M^−1^s^−1^ [75]	1.5 × 10^−1^ M [70]	2.1 × 10^4^ s^−1^
Threonine	4.6 × 10^4^ M^−1^s^−1^ [75]	9.8 × 10^−2^ M [70]	4.5 × 10^3^ s^−1^
Oxygen	2.2 × 10^9^ M^−1^s^−1^ [88]	6 × 10^−6^ M [89]	1.3 × 10^4^ s^−1^
**Liver tissue**, **Membrane Compartment**			
MUFA ^3,9^	1.6 × 10^5^ M^−1^s^−1^ [79]	1.8 × 10^−1^ M [90]	2.8 × 10^4^ s^−1^
PUFA ^3,4,9^	1.3 × 10^6^ M^−1^s^−1^ [66]	7.7 × 10^−1^ M [90]	1.0 × 10^6^ s^−1^
Ubiquinol ^5^	2.5 × 10^3^ M^−1^s^−1^ [84]	<1.2 × 10^−3^ M [95]	<3.0 × 10^0^ s^−1^
α-Tocopherol ^6^	6.2 × 10^4^ M^−1^s^−1^ [84]	1.5 × 10^−4^ M [92]	9.4 × 10^0^ s^−1^
Lycopene ^7,8^	1.6 × 10^9^ M^−1^s^−1^ [74]	<6.2 × 10^−4^ M [96]	<9.9 × 10^5^ s^−1^
Methionine	8 × 10^3^ M^−1^s^−1^ [75]	6.3 × 10^−2^ M [70]	5.0 × 10^2^ s^−1^
Phenylalanine	1.2 × 10^4^ M^−1^s^−1^ [75]	1.7 × 10^−1^ M [70]	2.0 × 10^3^ s^−1^
Serine	1.4 × 10^5^ M^−1^s^−1^ [75]	1.3 × 10^−1^ M [70]	1.8 × 10^4^ s^−1^
Threonine	4.6 × 10^4^ M^−1^s^−1^ [75]	1.1 × 10^−1^ M [70]	5.1 × 10^3^ s^−1^
Oxygen	2.2 × 10^9^ M^−1^s^−1^ [88]	2.4 × 10^−5^ M [89]	5.3 × 10^4^ s^−1^

^1^ Concentrations refer to total rat liver or rat liver mitochondria, if not otherwise specified. The following reference numbers were utilized for all calculations, which were performed as described [70]: hepatocyte volume: 4.9 × 10^−15^ m^3^ [97]; hepatocyte total membrane area: 1.1 × 10^−7^ m^2^ [97]; hepatocyte mitochondrion volume: 2.7 × 10^−19^ m^3^ [98]; hepatocyte mitochondrion inner membrane area: 6.5 × 10^−12^ m^2^ [98]. Hepatocytes were assumed to contain 23.7% mitochondria by volume [97]. The inner mitochondrial membrane was approximated to contain 60% lipid by area (and to represent 8.7% of the total mitochondrial volume [70]), all other membranes would contain 80% lipid by area (and would represent 7.8% of the total hepatocyte volume [97]), the remainder being protein [98]. The hydrophobic core thickness of lipid bilayers was adopted to be 3.6 × 10^−9^ m for all membrane types [99]. ^2^ The rate constant of glutathione was calculated as weighted mean of the rate constants of protonated glutathione (6 × 10^7^ M^−1^s^−1^ [75]) and deprotonated glutathione (5 × 10^8^ M^−1^s^−1^ [75]) assuming a mitochondrial pH = 8 and a glutathione pKs = 9 [100] as described before [70]. ^3^ Relative fatty acid compositions [90] were transformed into compartment-specific molar concentrations as detailed before [70]. ^4^ The term “PUFA concentration” here corresponds to the total concentration of reactive bisallylic positions as justified elsewhere [70]. ^5^ The rate constant of ubiquinol was approximated by the calculated rate constant of 2,3-dimethoxy-5-methyl-1,4-benzenediol (“ubiquinol-0”) in nonpolar solvents [84]. The concentration of ubiquinol in the inner mitochondrial membrane was calculated from the ratio ubiquinol:complex III = 100:3 [91], followed by transformation as described [70]. Extra-mitochondrial hepatic membrane ubiquinol was estimated from the published [95] ubiquinone:phospholipid molar ratio of different hepatic subfractions (upper limit: 1:500). ^6^ α-Tocopherol has the highest rate constant of the four tocopherols (α, β, γ, δ) [84] and is the most highly concentrated tocopherol in humans [101]; it may thus reflect the total reactivity of tocopherols is most mammalian settings. Tocopherol contents in subcellular tissue fractions [92] were transformed into compartment-specific molar concentrations as described [70], adopting a rat liver density of 1.05 g/mL. ^7^ An upper limit for the membrane lycopene concentration was estimated from the gross concentration of lycopene in total rat liver (50 nmol/g) after high-dose oral supplementation (5 g/kg for 8 weeks), which was more than 10,000-fold higher than without supplementation [96]. An even distribution of lycopene in the lipid bilayer compartment of all hepatic cells was assumed, despite the fact that most lycopene will be accumulating in stellate cells [102]. Hence, the given value is certainly a substantial overestimation and thus a rather theoretical upper limit for the maximum total effect of all carotenoids combined. Mitochondria actively degrade lycopene and other carotenoids in the inner membrane to lower their apparent toxicity in this compartment [103]. Hence, carotenoids may be essentially absent in the inner mitochondrial membrane [104], precluding their separate analysis. ^8^ The concentration of quercetin (including metabolites) was approximated by the human plasma level reached after oral supplementation (a 200 g onion meal) [94]. Mitochondrial or hepatic concentrations appear to be unknown. As quercetin is both rather rapidly reacting [84] and rather highly concentrated in plasma after consumption of a typical flavonoid-rich meal [105], it may well represent the overall thiyl radical scavenging activity of nutritional phenols in toto. ^9^ Extra-mitochondrial hepatic membrane fatty acid composition was modeled by a 1:1 mix of the smooth and rough endoplasmic reticulum fatty acid compositions taken from [90]. These membranes provide more than 90% of the non-mitochondrial membrane area in hepatocytes [97].

## References

[1] Moosmann B. (2021). Redox biochemistry of the genetic code. Trends Biochem. Sci..

[2] Granold M., Hajieva P., Toşa M.I., Irimie F.D., Moosmann B. (2018). Modern diversification of the amino acid repertoire driven by oxygen. Proc. Natl. Acad. Sci. USA.

[3] Moosmann B., Schindeldecker M., Hajieva P. (2020). Cysteine, glutathione and a new genetic code: Biochemical adaptations of the primordial cells that spread into open water and survived biospheric oxygenation. Biol. Chem..

[4] Longo L.M., Blaber M. (2012). Protein design at the interface of the pre-biotic and biotic worlds. Arch. Biochem. Biophys..

[5] Longo L.M., Lee J., Blaber M. (2013). Simplified protein design biased for prebiotic amino acids yields a foldable, halophilic protein. Proc. Natl. Acad. Sci. USA.

[6] Shibue R., Sasamoto T., Shimada M., Zhang B., Yamagishi A., Akanuma S. (2018). Comprehensive reduction of amino acid set in a protein suggests the importance of prebiotic amino acids for stable proteins. Sci. Rep..

[7] Solis A.D. (2019). Reduced alphabet of prebiotic amino acids optimally encodes the conformational space of diverse extant protein folds. BMC Evol. Biol..

[8] Yagi S., Padhi A.K., Vucinic J., Barbe S., Schiex T., Nakagawa R., Simoncini D., Zhang K.Y.J., Tagami S. (2021). Seven amino acid types suffice to create the core fold of RNA polymerase. J. Am. Chem. Soc..

[9] Ilardo M., Meringer M., Freeland S., Rasulev B., Cleaves H.J. (2015). Extraordinarily adaptive properties of the genetically encoded amino acids. Sci. Rep..

[10] Ilardo M., Bose R., Meringer M., Rasulev B., Grefenstette N., Stephenson J., Freeland S., Gillams R.J., Butch C.J., Cleaves H.J. (2019). Adaptive properties of the genetically encoded amino acid alphabet are inherited from its subsets. Sci. Rep..

[11] Mayer-Bacon C., Agboha N., Muscalli M., Freeland S. (2021). Evolution as a guide to designing *xeno* amino acid alphabets. Int. J. Mol. Sci..

[12] Wächtershäuser G. (1992). Groundworks for an evolutionary biochemistry: The iron-sulphur world. Prog. Biophys. Mol. Biol..

[13] Martin W., Russell M.J. (2003). On the origins of cells: A hypothesis for the evolutionary transitions from abiotic geochemistry to chemoautotrophic prokaryotes, and from prokaryotes to nucleated cells. Philos. Trans. R. Soc. Lond. B Biol. Sci..

[14] Martin W.F. (2020). Older than genes: The acetyl CoA pathway and origins. Front. Microbiol..

[15] Selles Vidal L., Kelly C.L., Mordaka P.M., Heap J.T. (2018). Review of NAD(P)H-dependent oxidoreductases: Properties, engineering and application. Biochim. Biophys. Acta Proteins Proteom..

[16] Trisolini L., Gambacorta N., Gorgoglione R., Montaruli M., Laera L., Colella F., Volpicella M., De Grassi A., Pierri C.L. (2019). FAD/NADH dependent oxidoreductases: From different amino acid sequences to similar protein shapes for playing an ancient function. J. Clin. Med..

[17] Nelson J.W., Breaker R.R. (2017). The lost language of the RNA world. Sci. Signal..

[18] Goldman A.D., Kacar B. (2021). Cofactors are remnants of life’s origin and early evolution. J. Mol. Evol..

[19] Stubbe J., van Der Donk W.A. (1998). Protein radicals in enzyme catalysis. Chem. Rev..

[20] Tommos C. (2022). Insights into the thermodynamics and kinetics of amino-acid radicals in proteins. Annu. Rev. Biophys..

[21] Hanschmann E.M., Godoy J.R., Berndt C., Hudemann C., Lillig C.H. (2013). Thioredoxins, glutaredoxins, and peroxiredoxins--molecular mechanisms and health significance: From cofactors to antioxidants to redox signaling. Antioxid. Redox Signal..

[22] Levine R.L., Mosoni L., Berlett B.S., Stadtman E.R. (1996). Methionine residues as endogenous antioxidants in proteins. Proc. Natl. Acad. Sci. USA.

[23] Lim J.M., Kim G., Levine R.L. (2019). Methionine in proteins: It’s not just for protein initiation anymore. Neurochem. Res..

[24] Bender A., Hajieva P., Moosmann B. (2008). Adaptive antioxidant methionine accumulation in respiratory chain complexes explains the use of a deviant genetic code in mitochondria. Proc. Natl. Acad. Sci. USA.

[25] Schindeldecker M., Moosmann B. (2015). Protein-borne methionine residues as structural antioxidants in mitochondria. Amino Acids.

[26] Knight R.D., Freeland S.J., Landweber L.F. (2001). Rewiring the keyboard: Evolvability of the genetic code. Nat. Rev. Genet..

[27] Sengupta S., Yang X., Higgs P.G. (2007). The mechanisms of codon reassignments in mitochondrial genetic codes. J. Mol. Evol..

[28] Graham J.B., Aguilar N.M., Dudley R., Gans C. (1995). Implications of the late Palaeozoic oxygen pulse for physiology and evolution. Nature.

[29] Och L.M., Shields-Zhou G.A. (2012). The neoproterozoic oxygenation event: Environmental perturbations and biogeochemical cycling. Earth Sci. Rev..

[30] Gray H.B., Winkler J.R. (2021). Functional and protective hole hopping in metalloenzymes. Chem. Sci..

[31] Teo R.D., Wang R., Smithwick E.R., Migliore A., Therien M.J., Beratan D.N. (2019). Mapping hole hopping escape routes in proteins. Proc. Natl. Acad. Sci. USA.

[32] Gray H.B., Winkler J.R. (2018). Living with oxygen. Acc. Chem. Res..

[33] Saxena C., Sancar A., Zhong D. (2004). Femtosecond dynamics of DNA photolyase: Energy transfer of antenna initiation and electron transfer of cofactor reduction. J. Phys. Chem. B.

[34] Gray H.B., Winkler J.R. (2015). Hole hopping through tyrosine/tryptophan chains protects proteins from oxidative damage. Proc. Natl. Acad. Sci. USA.

[35] Moosmann B., Behl C. (2000). Cytoprotective antioxidant function of tyrosine and tryptophan residues in transmembrane proteins. Eur. J. Biochem..

[36] Hajieva P., Bayatti N., Granold M., Behl C., Moosmann B. (2015). Membrane protein oxidation determines neuronal degeneration. J. Neurochem..

[37] Moosmann B., Behl C. (2002). Secretory peptide hormones are biochemical antioxidants: Structure-activity relationship. Mol. Pharmacol..

[38] Cardona T. (2019). Thinking twice about the evolution of photosynthesis. Open Biol..

[39] Cardona T., Sanchez-Baracaldo P., Rutherford A.W., Larkum A.W. (2019). Early Archean origin of Photosystem II. Geobiology.

[40] Fournier G.P., Moore K.R., Rangel L.T., Payette J.G., Momper L., Bosak T. (2021). The Archean origin of oxygenic photosynthesis and extant cyanobacterial lineages. Proc. Biol. Sci..

[41] Oliver T., Sanchez-Baracaldo P., Larkum A.W., Rutherford A.W., Cardona T. (2021). Time-resolved comparative molecular evolution of oxygenic photosynthesis. Biochim. Biophys. Acta Bioenerg..

[42] Boden J.S., Konhauser K.O., Robbins L.J., Sanchez-Baracaldo P. (2021). Timing the evolution of antioxidant enzymes in cyanobacteria. Nat. Commun..

[43] Jablonska J., Tawfik D.S. (2021). The evolution of oxygen-utilizing enzymes suggests early biosphere oxygenation. Nat. Ecol. Evol..

[44] Crowe S.A., Dossing L.N., Beukes N.J., Bau M., Kruger S.J., Frei R., Canfield D.E. (2013). Atmospheric oxygenation three billion years ago. Nature.

[45] Planavsky N.J., Asael D., Hofmann A., Reinhard C.T., Lalonde S.V., Knudsen A., Wang X., Ossa Ossa F., Pecoits E., Smith A.J. (2014). Evidence for oxygenic photosynthesis half a billion years before the great oxidation event. Nat. Geosci..

[46] Lalonde S.V., Konhauser K.O. (2015). Benthic perspective on Earth’s oldest evidence for oxygenic photosynthesis. Proc. Natl. Acad. Sci. USA.

[47] Frei R., Crowe S.A., Bau M., Polat A., Fowle D.A., Dossing L.N. (2016). Oxidative elemental cycling under the low O_2_ Eoarchean atmosphere. Sci. Rep..

[48] He H., Wu X., Xian H., Zhu J., Yang Y., Lv Y., Li Y., Konhauser K.O. (2021). An abiotic source of Archean hydrogen peroxide and oxygen that pre-dates oxygenic photosynthesis. Nat. Commun..

[49] Labunskyy V.M., Hatfield D.L., Gladyshev V.N. (2014). Selenoproteins: Molecular pathways and physiological roles. Physiol. Rev..

[50] Mukai T., Englert M., Tripp H.J., Miller C., Ivanova N.N., Rubin E.M., Kyrpides N.C., Söll D. (2016). Facile recoding of selenocysteine in nature. Angew. Chem. Int. Ed. Engl..

[51] Vieira-Silva S., Rocha E.P. (2008). An assessment of the impacts of molecular oxygen on the evolution of proteomes. Mol. Biol. Evol..

[52] Giles G.I., Jacob C. (2002). Reactive sulfur species: An emerging concept in oxidative stress. Biol. Chem..

[53] Zeida A., Guardia C.M., Lichtig P., Perissinotti L.L., Defelipe L.A., Turjanski A., Radi R., Trujillo M., Estrin D.A. (2014). Thiol redox biochemistry: Insights from computer simulations. Biophys. Rev..

[54] Trifonov E.N. (2009). The origin of the genetic code and of the earliest oligopeptides. Res. Microbiol..

[55] Zhao M., Ding R., Liu Y., Ji Z., Zhao Y. (2022). Determination of the amino acid recruitment order in early life by genome-wide analysis of amino acid usage bias. Biomolecules.

[56] Sevier C.S., Kaiser C.A. (2002). Formation and transfer of disulphide bonds in living cells. Nat. Rev. Mol. Cell Biol..

[57] Giles N.M., Watts A.B., Giles G.I., Fry F.H., Littlechild J.A., Jacob C. (2003). Metal and redox modulation of cysteine protein function. Chem. Biol..

[58] Lu J., Holmgren A. (2014). The thioredoxin antioxidant system. Free Radic. Biol. Med..

[59] Moosmann B., Behl C. (2008). Mitochondrially encoded cysteine predicts animal lifespan. Aging Cell.

[60] Chang R.L., Stanley J.A., Robinson M.C., Sher J.W., Li Z., Chan Y.A., Omdahl A.R., Wattiez R., Godzik A., Matallana-Surget S. (2020). Protein structure, amino acid composition and sequence determine proteome vulnerability to oxidation-induced damage. EMBO J..

[61] Schindeldecker M., Stark M., Behl C., Moosmann B. (2011). Differential cysteine depletion in respiratory chain complexes enables the distinction of longevity from aerobicity. Mech. Ageing Dev..

[62] Moosmann B. (2011). Respiratory chain cysteine and methionine usage indicate a causal role for thiyl radicals in aging. Exp. Gerontol..

[63] Giles G.I., Nasim M.J., Ali W., Jacob C. (2017). The reactive sulfur species concept: 15 years on. Antioxidants.

[64] Marino S.M., Gladyshev V.N. (2010). Cysteine function governs its conservation and degeneration and restricts its utilization on protein surfaces. J. Mol. Biol..

[65] Marino S.M., Gladyshev V.N. (2012). Analysis and functional prediction of reactive cysteine residues. J. Biol. Chem..

[66] Schöneich C., Asmus K.D., Dillinger U., von Bruchhausen F. (1989). Thiyl radical attack on polyunsaturated fatty acids: A possible route to lipid peroxidation. Biochem. Biophys. Res. Commun..

[67] Chatgilialoglu C., Altieri A., Fischer H. (2002). The kinetics of thiyl radical-induced reactions of monounsaturated fatty acid esters. J. Am. Chem. Soc..

[68] Mozziconacci O., Kerwin B.A., Schöneich C. (2010). Reversible hydrogen transfer between cysteine thiyl radical and glycine and alanine in model peptides: Covalent H/D exchange, radical-radical reactions, and L- to D-Ala conversion. J. Phys. Chem. B.

[69] Schöneich C. (2008). Mechanisms of protein damage induced by cysteine thiyl radical formation. Chem. Res. Toxicol..

[70] Kunath S., Schindeldecker M., De Giacomo A., Meyer T., Sohre S., Hajieva P., von Schacky C., Urban J., Moosmann B. (2020). Prooxidative chain transfer activity by thiol groups in biological systems. Redox Biol..

[71] Heymans V., Kunath S., Hajieva P., Moosmann B. (2021). Cell culture characterization of prooxidative chain-transfer agents as novel cytostatic drugs. Molecules.

[72] Smith W.V. (1946). Regulator theory in emulsion polymerization; chain transfer of low molecular weight mercaptans in emulsion and oil-phase. J. Am. Chem. Soc..

[73] Smith W.V. (1946). Regulator theory in emulsion polymerization; control of reaction rate by diffusion for high molecular weight mercaptans. J. Am. Chem. Soc..

[74] Mortensen A., Skibsted L.H., Sampson J., Rice-Evans C., Everett S.A. (1997). Comparative mechanisms and rates of free radical scavenging by carotenoid antioxidants. FEBS Lett..

[75] Nauser T., Pelling J., Schöneich C. (2004). Thiyl radical reaction with amino acid side chains: Rate constants for hydrogen transfer and relevance for posttranslational protein modification. Chem. Res. Toxicol..

[76] Tartaro Bujak I., Mihaljevic B., Ferreri C., Chatgilialoglu C. (2016). The influence of antioxidants in the thiyl radical induced lipid peroxidation and geometrical isomerization in micelles of linoleic acid. Free Radic. Res..

[77] Stoyanovsky D.A., Maeda A., Atkins J.L., Kagan V.E. (2011). Assessments of thiyl radicals in biosystems: Difficulties and new applications. Anal. Chem..

[78] Chatgilialoglu C., Zambonin L., Altieri A., Ferreri C., Mulazzani Q.G., Landi L. (2002). Geometrical isomerism of monounsaturated fatty acids: Thiyl radical catalysis and influence of antioxidant vitamins. Free Radic. Biol. Med..

[79] Chatgilialoglu C., Ferreri C. (2005). Trans lipids: The free radical path. Acc. Chem. Res..

[80] Zambonin L., Ferreri C., Cabrini L., Prata C., Chatgilialoglu C., Landi L. (2006). Occurrence of trans fatty acids in rats fed a trans-free diet: A free radical-mediated formation?. Free Radic. Biol. Med..

[81] Melchiorre M., Torreggiani A., Chatgilialoglu C., Ferreri C. (2011). Lipid markers of “geometrical” radical stress: Synthesis of monotrans cholesteryl ester isomers and detection in human plasma. J. Am. Chem. Soc..

[82] Denisov E., Chatgilialoglu C., Shestakov A., Denisova T. (2009). Rate constants and transition-state geometry of reactions of alkyl, alkoxyl, and peroxyl radicals with thiols. Int. J. Chem. Kinet..

[83] Yin H., Xu L., Porter N.A. (2011). Free radical lipid peroxidation: Mechanisms and analysis. Chem. Rev..

[84] Denisova T.G., Denisov E.T. (2009). Reactivity of natural phenols in radical reactions. Kinet. Catal..

[85] Trachootham D., Alexandre J., Huang P. (2009). Targeting cancer cells by ROS-mediated mechanisms: A radical therapeutic approach?. Nat. Rev. Drug Discov..

[86] Gorrini C., Harris I.S., Mak T.W. (2013). Modulation of oxidative stress as an anticancer strategy. Nat. Rev. Drug Discov..

[87] Li X., Cobb C.E., Hill K.E., Burk R.F., May J.M. (2001). Mitochondrial uptake and recycling of ascorbic acid. Arch. Biochem. Biophys..

[88] Schöneich C. (2016). Thiyl radicals and induction of protein degradation. Free Radic. Res..

[89] Jones D.P. (1984). Effect of mitochondrial clustering on O2 supply in hepatocytes. Am. J. Physiol..

[90] Luit H., Berger R., Hommes F.A. (1975). Fatty acid composition of some cellular membranes of fetal rat liver. Biol. Neonate.

[91] Lenaz G., Genova M.L. (2009). Structural and functional organization of the mitochondrial respiratory chain: A dynamic super-assembly. Int. J. Biochem. Cell Biol..

[92] Buttriss J.L., Diplock A.T. (1988). The relationship between alpha-tocopherol and phospholipid fatty acids in rat liver subcellular membrane fractions. Biochim. Biophys. Acta.

[93] Mikasa H., Ageta T., Mizoguchi N., Kodama H. (1982). Determination of glutathione and glutathione disulfide in rat tissues using isotachophoretic analyzer. Anal. Biochem..

[94] Day A.J., Mellon F., Barron D., Sarrazin G., Morgan M.R., Williamson G. (2001). Human metabolism of dietary flavonoids: Identification of plasma metabolites of quercetin. Free Radic. Res..

[95] Teclebrhan H., Jakobsson-Borin A., Brunk U., Dallner G. (1995). Relationship between the endoplasmic reticulum-Golgi membrane system and ubiquinone biosynthesis. Biochim. Biophys. Acta.

[96] Boileau T.W., Clinton S.K., Erdman J.W. (2000). Tissue lycopene concentrations and isomer patterns are affected by androgen status and dietary lycopene concentration in male F344 rats. J. Nutr..

[97] Weibel E.R., Stäubli W., Gnägi H.R., Hess F.A. (1969). Correlated morphometric and biochemical studies on the liver cell. I. Morphometric model, stereologic methods, and normal morphometric data for rat liver. J. Cell Biol..

[98] Schwerzmann K., Cruz-Orive L.M., Eggman R., Sänger A., Weibel E.R. (1986). Molecular architecture of the inner membrane of mitochondria from rat liver: A combined biochemical and stereological study. J. Cell Biol..

[99] Nagle J.F., Tristram-Nagle S. (2000). Structure of lipid bilayers. Biochim. Biophys. Acta.

[100] Nauser T., Koppenol W.H., Schöneich C. (2015). Protein thiyl radical reactions and product formation: A kinetic simulation. Free Radic. Biol. Med..

[101] Traber M.G. (2007). Vitamin E regulatory mechanisms. Annu. Rev. Nutr..

[102] Teodoro A.J., Perrone D., Martucci R.B., Borojevic R. (2009). Lycopene isomerisation and storage in an in vitro model of murine hepatic stellate cells. Eur. J. Nutr..

[103] Amengual J., Lobo G.P., Golczak M., Li H.N., Klimova T., Hoppel C.L., Wyss A., Palczewski K., von Lintig J. (2011). A mitochondrial enzyme degrades carotenoids and protects against oxidative stress. FASEB J..

[104] Palczewski G., Amengual J., Hoppel C.L., von Lintig J. (2014). Evidence for compartmentalization of mammalian carotenoid metabolism. FASEB J..

[105] Mannen R., Yasuda M.T., Sano A., Goda T., Shimoi K., Ichikawa Y. (2019). Changes in plasma concentration of flavonoids after ingestion of a flavonoid-rich meal prepared with basic foodstuffs. Funct. Foods Health Dis..

[106] Francoleon N.E., Carrington S.J., Fukuto J.M. (2011). The reaction of H(2)S with oxidized thiols: Generation of persulfides and implications to H(2)S biology. Arch. Biochem. Biophys..

[107] Bianco C.L., Chavez T.A., Sosa V., Saund S.S., Nguyen Q.N.N., Tantillo D.J., Ichimura A.S., Toscano J.P., Fukuto J.M. (2016). The chemical biology of the persulfide (RSSH)/perthiyl (RSS·) redox couple and possible role in biological redox signaling. Free Radic. Biol. Med..

[108] Traber M.G., Atkinson J. (2007). Vitamin E, antioxidant and nothing more. Free Radic. Biol. Med..

[109] Ohlow M.J., Granold M., Schreckenberger M., Moosmann B. (2012). Is the chromanol head group of vitamin E nature’s final truth on chain-breaking antioxidants?. FEBS Lett..

[110] Denisov E.T., Denisova T.G. (2009). The reactivity of natural phenols. Russ. Chem. Rev..

[111] Hung W.L., Ho C.T., Hwang L.S. (2011). Inhibitory activity of natural occurring antioxidants on thiyl radical-induced trans-arachidonic acid formation. J. Agric. Food Chem..

[112] Akaike T., Ida T., Wei F.Y., Nishida M., Kumagai Y., Alam M.M., Ihara H., Sawa T., Matsunaga T., Kasamatsu S. (2017). Cysteinyl-tRNA synthetase governs cysteine polysulfidation and mitochondrial bioenergetics. Nat. Commun..

[113] Fukuto J.M., Ignarro L.J., Nagy P., Wink D.A., Kevil C.G., Feelisch M., Cortese-Krott M.M., Bianco C.L., Kumagai Y., Hobbs A.J. (2018). Biological hydropersulfides and related polysulfides—A new concept and perspective in redox biology. FEBS Lett..

[114] Fujii S., Sawa T., Motohashi H., Akaike T. (2019). Persulfide synthases that are functionally coupled with translation mediate sulfur respiration in mammalian cells. Br. J. Pharmacol..

[115] Burger N., James A.M., Mulvey J.F., Hoogewijs K., Ding S., Fearnley I.M., Loureiro-Lopez M., Norman A.A.I., Arndt S., Mottahedin A. (2022). ND3 Cys39 in complex I is exposed during mitochondrial respiration. Cell Chem. Biol..

[116] Ida T., Sawa T., Ihara H., Tsuchiya Y., Watanabe Y., Kumagai Y., Suematsu M., Motohashi H., Fujii S., Matsunaga T. (2014). Reactive cysteine persulfides and S-polythiolation regulate oxidative stress and redox signaling. Proc. Natl. Acad. Sci. USA.

[117] Qabazard B., Li L., Gruber J., Peh M.T., Ng L.F., Kumar S.D., Rose P., Tan C.H., Dymock B.W., Wei F. (2014). Hydrogen sulfide is an endogenous regulator of aging in Caenorhabditis elegans. Antioxid. Redox Signal..

[118] Sokolov A.S., Nekrasov P.V., Shaposhnikov M.V., Moskalev A.A. (2021). Hydrogen sulfide in longevity and pathologies: Inconsistency is malodorous. Ageing Res. Rev..

[119] Kunath S., Moosmann B. (2020). What is the rate-limiting step towards aging? Chemical reaction kinetics might reconcile contradictory observations in experimental aging research. Geroscience.

[120] Moosmann B. (2021). Flux control in the aging cascade. Aging.

[121] Gromer S., Johansson L., Bauer H., Arscott L.D., Rauch S., Ballou D.P., Williams CHJr Schirmer R.H., Arner E.S. (2003). Active sites of thioredoxin reductases: Why selenoproteins?. Proc. Natl. Acad. Sci. USA.

[122] Nauser T., Steinmann D., Koppenol W.H. (2012). Why do proteins use selenocysteine instead of cysteine?. Amino Acids.

[123] Taskov K., Chapple C., Kryukov G.V., Castellano S., Lobanov A.V., Korotkov K.V., Guigo R., Gladyshev V.N. (2005). Nematode selenoproteome: The use of the selenocysteine insertion system to decode one codon in an animal genome?. Nucleic Acids Res..

[124] Nauser T., Steinmann D., Grassi G., Koppenol W.H. (2014). Why selenocysteine replaces cysteine in thioredoxin reductase: A radical hypothesis. Biochemistry.

[125] Snider G.W., Ruggles E., Khan N., Hondal R.J. (2013). Selenocysteine confers resistance to inactivation by oxidation in thioredoxin reductase: Comparison of selenium and sulfur enzymes. Biochemistry.

[126] Copley S.D., Dhillon J.K. (2002). Lateral gene transfer and parallel evolution in the history of glutathione biosynthesis genes. Genome Biol..

[127] Gest N., Gautier H., Stevens R. (2013). Ascorbate as seen through plant evolution: The rise of a successful molecule?. J. Exp. Bot..

[128] Zhao R., Lind J., Merenyi G., Eriksen T.E. (1997). Significance of the intramolecular transformation of glutathione thiyl radicals to α-aminoalkyl radicals. Thermochemical and biological implications. J. Chem. Soc. Perkin Trans..

[129] Mozziconacci O., Williams T.D., Schöneich C. (2012). Intramolecular hydrogen transfer reactions of thiyl radicals from glutathione: Formation of carbon-centered radical at Glu, Cys, and Gly. Chem. Res. Toxicol..

[130] Denes F., Pichowicz M., Povie G., Renaud P. (2014). Thiyl radicals in organic synthesis. Chem. Rev..

